# 794. The Role of the Environment in Healthcare-associated Transmission of Vancomycin Resistant Enterococcus: A Proof of Concept Study

**DOI:** 10.1093/ofid/ofab466.990

**Published:** 2021-12-04

**Authors:** Amber L Linkneheld-Struk, Victoria R Williams, Lorraine Maze Dit Mieusement, Natasha Salt, Adrienne Chan, Jerome A Leis

**Affiliations:** 1 Sunnybrook Health Sciences Centre, Pickering, Ontario, Canada; 2 Sunnybrook Health Sciences Centre, Toronto, ON, Toronto, ON, Canada

## Abstract

**Background:**

Transmission of Vancomycin Resistant Enterococcus (VRE) from environment to patient and patient to patient can both occur in healthcare settings. Due to the COVID-19 pandemic, a cohort of exposed patients on an inpatient unit with an extensive VRE outbreak needed to switch physical locations with a non-exposed patient population. By comparing outcomes of both cohorts, we aimed to determine the role of the physical environment (both direct and indirect contact) as compared to the patient population, in ongoing VRE transmission.

**Methods:**

From 10 March to 21 April 2021, 41 new nosocomial acquisitions of VRE were detected as part of a VRE outbreak on a 34-bed acute care unit. Prior to the switch of units, extensive cleaning of the unit was conducted including electrostatic adjuncts to standard cleaning and environmental swabbing for VRE yielded no positive surfaces. The exposed cohort included 3 of 30 patients with VRE while the non-exposed cohort had 0 of 28 VRE positive patients based on prevalence testing on 21 April 2021. Following the physical relocation of both cohorts on 22 April, 2021, prospective VRE screening was performed on both units for one month including on admission, discharge and weekly prevalence screening. Hand hygiene compliance rates on both units was measured using group electronic monitoring.

**Results:**

Figure 1 depicts the timeline and number of VRE cases before and after the unit switch. Following relocation of the VRE exposed cohort to the new unit, no further VRE transmission was detected (0/235 VRE screens; 0 VRE cases per 1000 patient days). Conversely, there were new VRE transmissions (3/99 VRE screens, 5 VRE cases per 1000 patient days) in the non-exposed cohort. When the units resumed their original location, one additional case of VRE was identified in the exposed cohort upon return to their original location. These transmissions occurred despite HH compliance of 94% (141,610/150,706) during the entire study period on the outbreak unit, which was consistently higher than on the non-outbreak unit (141,589/227,136, 62%).Figure 1.

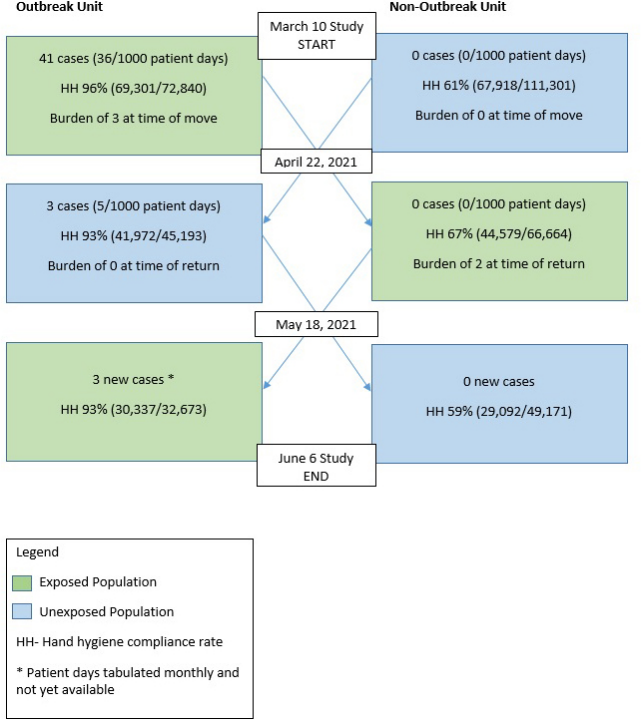

**Conclusion:**

The environmental reservoir for VRE may be more important in transmission than the patient reservoir. These findings underscore the importance of environmental cleaning to contain VRE outbreaks.

**Disclosures:**

**All Authors**: No reported disclosures

